# Steady Increment of Immature Platelet Fraction Is Suppressed by Irradiation in Single-Donor Platelet Components during Storage

**DOI:** 10.1371/journal.pone.0085465

**Published:** 2014-01-08

**Authors:** Hong Hong, Wenbin Xiao, Robert W. Maitta

**Affiliations:** 1 Department of Pathology, University Hospitals Case Medical Center, Cleveland, Ohio, United States of America; 2 Case Western Reserve University School of Medicine, Cleveland, Ohio, United States of America; University of Leuven, Belgium

## Abstract

Circulating immature platelet fraction (IPF) reflects real-time thrombopoiesis and correlates with platelet recovery from thrombocytopenic presentations. To understand the dynamics of IPF in platelet transfusions, we quantified the %-IPF in single-donor platelet components (SDP) during prolonged storage. %-IPF significantly increased from baseline by day 5 post-donation. Absolute IPF counts (A-IPC) had similar significant increments. However, gamma-irradiation suppressed the increments of %-IPF and A-IPC by >50%. Ultrastructural analysis of SDP units at day 10 showed well preserved morphology of immature platelets. Our findings suggest that IPF might actively expand ex-vivo and may have a longer shelf life than their mature counterparts. Closer study of IPF may be of critical clinical importance for transfusion practices.

## Introduction

Circulating immature platelets are newly released from the bone marrow, contain higher concentration of RNA than mature platelets, and are measured by automated hematology analyzers equipped with a reticulocyte detection channel as the percent immature platelet fraction (%-IPF) [1]. A high %-IPF characterizes either consumptive or recovering thrombocytopenic disorders [2], while low %-IPF is characteristic of bone marrow suppression states [3]. However, the absolute immature platelet count (A-IPC) calculated from %-IPF may better reflect real-time thrombopoiesis [4]; and correlate with platelet recovery in conditions such as immune thrombocytopenic purpura and post-chemotherapy thrombocytopenia [1], [2].

The clinical significance of %-IPF and A-IPC in platelet transfusion has not been elucidated. A recent report showed that transfusion of hematopoietic-progenitor-cell transplanted children with IPF-rich platelet units significantly reduced the overall number of platelet transfusions required by patients compared to those receiving IPF-poor units [5]. Even though this study presented data from a small pediatric oncology patient cohort, it suggested that larger randomized trials should be performed to address if the concentration of immature platelets in a platelet product can better benefit patients requiring transfusion support. Recently, increments in IPF during storage in platelet units have been reported but were believed to be non-specific findings with no biological significance due to the nature of the analyzer used [6]. In light of these contradicting reports, to better understand the IPF/A-IPC in platelet components, and to determine their dynamics during storage we measured the %-IPF, calculated the A-IPC, and performed immature platelet ultrastructure analysis in SDP units maintained for an extended storage period.

## Materials and Methods

### Platelets

SDP specimens were obtained from our local blood suppliers: American Red Cross (Cleveland, OH, USA) and LifeShare Community Blood Services (Lorain, OH, USA). All units were from healthy volunteers, leukoreduced, collected by apheresis, stored in PVC bags (Fenwal Inc., Lake Zurich, IL, USA) with ACD-A (10–15%) as anticoagulation [7], and with similar platelet counts as established by the Food and Drug Administration's Code of Federal Regulations Title 21. Units were kept at room temperature (22–24°C) under continuous gentle agitation [8]. Aliquots (1 mL) were taken from specimens on day of donation (day 0) and 3, 5 and 7 days post-donation. Separately, we followed 5 SDP specimens over a two-week period sampling them 3, 5, 7, 10 and 14 days post-donation. Lastly, 1 mL aliquots of 12 SDP specimens that were randomized into two groups on day of donation, one group receiving gamma-irradiation (30 Gy) using the IBL437C irradiator (CIS Biointernational, Bedford, MA) according to the manufacturer's protocol on day 0 while the other group did not, were sampled on day 5.

### Platelet counts, IPF analysis and calculations

All aliquots were tested for optical platelet (fluorescent) count, mean platelet volume (MPV), platelet-large cell ratio (P-LCR), %-IPF and %-High (H)-IPF within 1 hour of sampling using the Sysmex hematology analyzer XE-5000 according to manufacturer's protocols (Sysmex America Inc., Mundelein, IL). Sysmex e-Check (XE)^TM^ controls (Sysmex America Inc., Mundelein, IL) were measured after the analysis of every 50 samples. XbarM control program was also applied to re-assure the equipment's quality control as recommended by the manufacturer (Sysmex America Inc, Mundelein, IL). A-IPC and H-IPF counts (H-IPC) were obtained by multiplying the %-IPF or %-H-IPF times the optical platelet count respectively.

### Transmission electron microscopy

Three 1 mL aliquots from day 3 and three from day 10 were fixed by immersion in quarter-strength Karnovsky fixative solution for 2 hours at room temperature. After washing, specimens were post-fixed for 2 hours in an unbuffered 1:1 mixture of 2% osmium tetroxide and 3% potassium ferrocyanide. After rinsing with distilled water, specimens were soaked overnight in an acidified solution of 0.25% uranyl acetate. Specimens were then rinsed in distilled water, dehydrated in ascending concentrations of ethanol, passed through propylene oxide, and embedded in a Poly/Bed 812 embedding media (Polysciences, Warrington, PA). Thin sections were cut on a RMC MT6000-XL ultramicrotome (Boeckeler Instruments, Inc., Tucson, AZ). These were mounted on Gilder square 300 mesh nickel grids (Electron Microscopy Sciences, Hatfield, PA) and sequentially stained with acidified methanolic uranyl acetate and stable lead staining solution. Sections were subsequently coated on a Denton DV-401 carbon coater (Denton Vacuum LLC, Moorestown, NJ), and examined in a JEOL 1200EX electron microscope (JEOL., Ltd., Tokyo, Japan).

### Statistical analysis

All statistics were performed using Prism 6 (GraphPad Software Inc., La Jolla, CA). Results are presented as mean ± SD unless otherwise specified. Intergroup data comparisons were performed using the Student *t* test. All *p* values are two-sided with a type I error rate of 5% and a p<0.05 set for significance.

## Results

**Figure 1:**
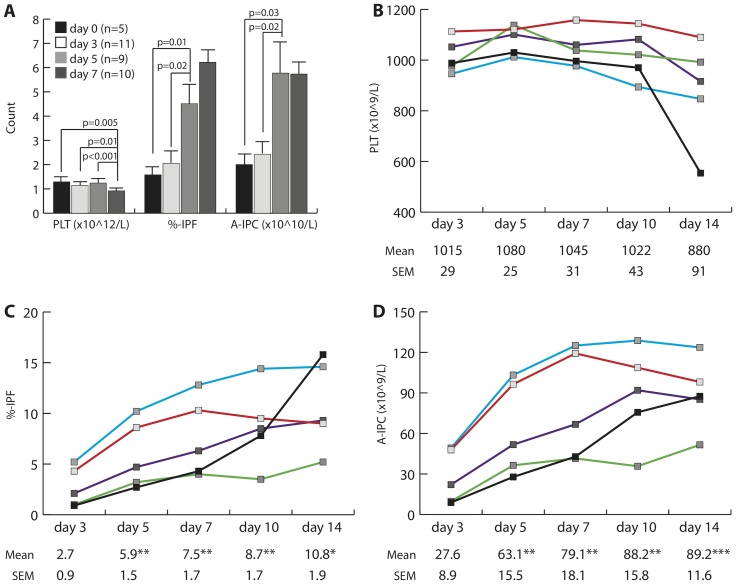
Steady increment of IPF and A-IPC in SDP during storage. **A**, Platelet count, %-IPF and A-IPC of different SDP on day 0, 3, 5 and 7 post donation during storage; N represents the number of SDP specimens measured experimentally. **B**–**D**, Serial monitoring of platelet parameters in SDP during storage including platelet count (**B**), %-IPF (**C**) and A-IPC (**D**) on day 3, 5, 7, 10, and 14 (N = 5). * p<0.05, ** p<0.005, *** p<0.001 when compared to day 3. Both Mean and SEM are reported for each specimen per time point.

%-IPF on day 0 was 1.58±0.3% similar to the reference range we established for the Sysmex XE-5000 (2.9±1.4%, range 0.8–5.7%, N = 40) which correlates with ranges reported for other analyzers [1], [9], [10]; A-IPC (×10^9^/L) was 20.0±4.0. As shown in Figure 1A, optical platelet counts (×10^9^/L) were stable and did not significantly decrease until day 7 (day 0, 1290.2±125.5 vs. day 7, 921.6±57.3, *p* = 0.005). On the contrary, %-IPF and A-IPC (×10^9^/L) significantly increased by day 5 compared to day 0 (4.5±0.8% and 57.7±12.5, *p* = 0.01 and *p* = 0.03 respectively) and %-IPF further increased on day 7 while A-IPC seemed to reach a plateau (6.2±0.5% and 57.4±5.2, respectively).

To test if the %-IPF/A-IPC increment in storage was due to random variation among units or A-IPC expansion ex-vivo, we followed %-IPF and A-IPC changes of SDP from five donors for 14 days. As shown in Figure 1B, optical platelet counts of the five units were not significantly different until day 14. As in the previous results for random units, both %-IPF and A-IPC had significant increases on day 5 and remained significantly high thereafter (Figures 1C and 1D). Similarly, while %-IPF continued to increase from days 7 to 14, just as before A-IPC reached a plateau remaining high for the duration of the experiments.

**Figure 2:**
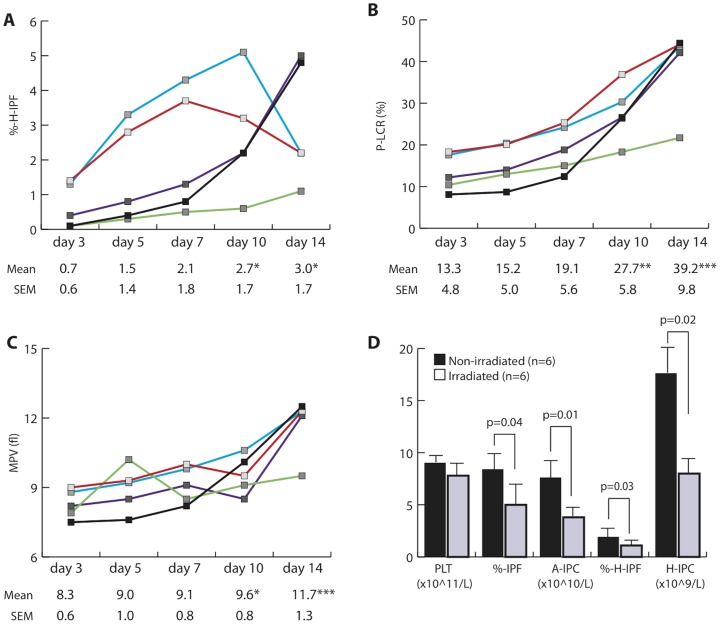
A–C, Serial monitoring of platelet parameters in SDP during storage (A, %-H-IPF; B, P-LCR; C, MPV, N = 5). * p<0.05, ** p<0.005, *** p<0.001 when compared to day 3. Both Mean and SEM are reported for each specimen per time point. **D**, Platelet parameters on day 5 post donation in response to gamma-irradiation; N = 6/group.

H-IPF represents the fraction of immature platelets within the IPF with the highest RNA content [11]. As shown in Figure 2A, both %-H-IPF and H-IPC (×10^9^/L) in the five SDPs also increased by day 10 (2.7±1.7% vs. 0.7±0.6% on day 3, *p* = 0.036; and 27±16 vs. 8.5±8.3 on day 3, *p* = 0.04; respectively). Analysis of additional parameters obtained with each run showed significant increments of MPV and P-LCR (Figures 2B and 2C). P-LCR represents the percentage of giant platelets with a volume >12 fL; these were significantly increased by day 10 (Figure 2B). Consistent with this increase of giant platelets, MPV demonstrated similar increases on days 10 and 14. (Figure 2C). To exclude non-specific staining such as platelet aggregates formation in product units, we reviewed the flow histograms of all samples and determined that all showed a normal platelet distribution (data not shown).

To analyze if the apparent increases in immature platelets was linked to biosynthetically intact platelet machinery, we analyzed the effects of irradiation on %-IPF/A-IPC and %-H-IPF/H-IPC of 12 SDP specimens randomly divided into irradiated and non-irradiated groups. On days 0 and 3 platelet count, %-IPF, A-IPC, %-H-IPF, H-IPC, P-LCR, and MPV were comparable between the two groups (data not shown). On day 5 irradiation did not affect platelet counts; however, %-IPF, A-IPC, %-H-IPF, and H-IPC were significantly reduced by 50% in the irradiated group (Figure 2D).

**Figure 3:**
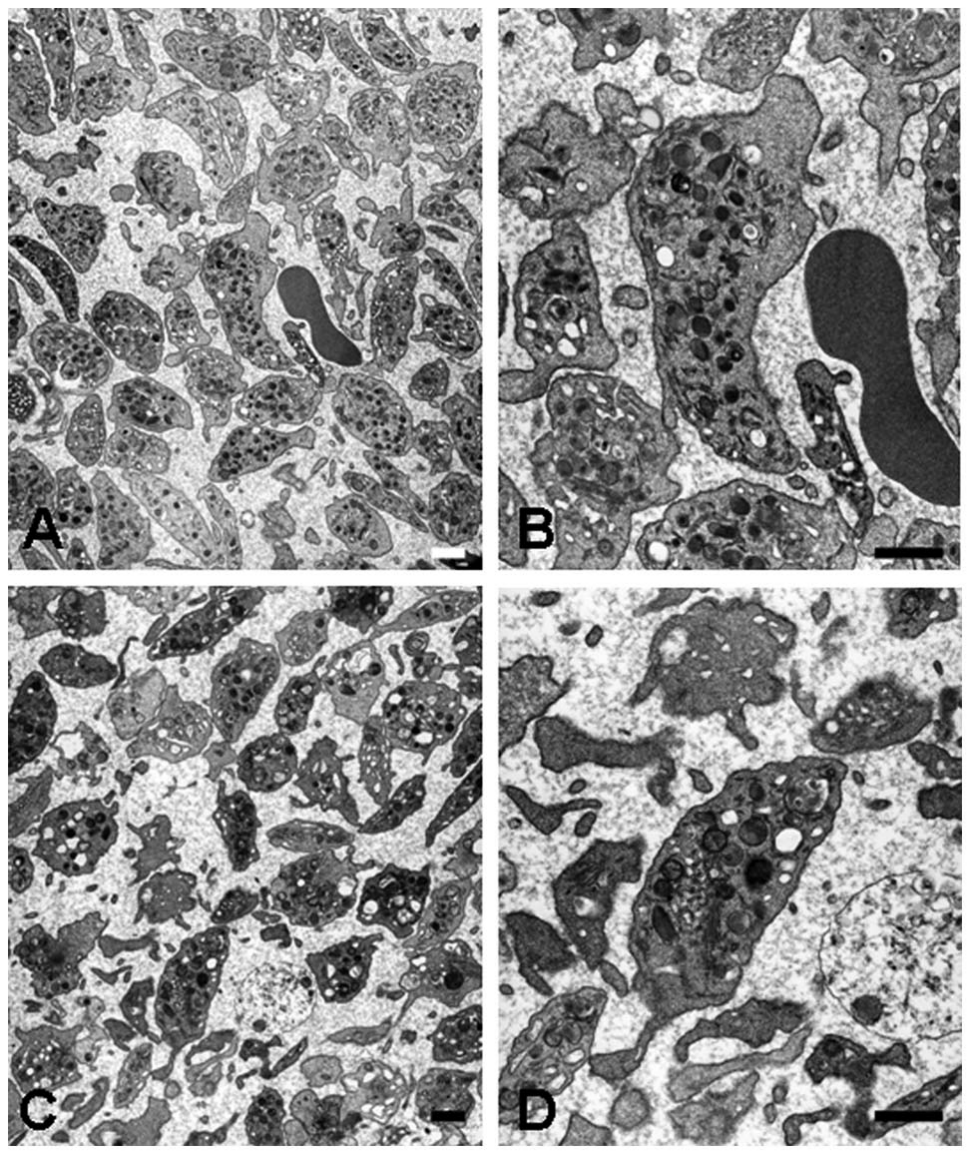
A–D, Transmission Electron Microscopy (TEM) analysis of platelet morphology in SDP units during prolonged storage. A, Representative image of day 3 platelets and immature platelets in SDP; scale bar indicates 2 µm. B, Cross-sectional image of the immature platelet on day 3 as shown in Fig 3A with an adjacent erythrocyte for size comparison. The immature platelets contains glycogen, mitochondria, dense granules, alpha granules, and open canalicular system; scale bar indicates 500 nm. C, Representative image of the cross-sectional image of platelet in SDP on day 10; scale bar indicates 2 µm. D, Cross-sectional image of the immature platelet on day 10 as shown in Fig 3C; scale bar indicates 500 nm.

To understand IPF morphology in SDP during storage, we performed SDP ultrastructural analysis focusing on the internal structure and granule integrity of immature platelets using transmission electron microscopy. As shown in Figure 3A–B, at day 3 immature platelets appear intact and larger than a comparable normal erythrocyte. Immature platelets appeared to have a larger number of well-preserved glycogen, mitochondria, dense granules, alpha granules, and open canalicular system than mature platelets in the fields analyzed. At day 10 immature platelets still appeared mostly morphologically intact; however, there was evident mature platelet death since membrane ghosts could be seen more commonly in these samples (Figure 3C–D).

## Discussion

Together %-IPF and %-H-IPF increments in the same units followed for two-weeks suggest ex-vivo expansion as a plausible explanation. A recent report indicated that the increment of %-IPF in storage represents non-specific artifacts of platelets degranulation [6]. This report, however, utilized an analyzer without the optical channel which can help rule out interference from fragmentocytes and/or platelet aggregates. Platelet counts and %-IPF in the analyzer utilized in our study depend on both fluo­rescence activation and platelet volume (optical analysis) which minimizes influences from non-specific staining of a myriad of granules [12]. Similarly, the flow histograms of all analyzed samples showing a normal platelet distribution argue against the artifactual nature of our measurements or the predominant presence of platelet aggregates as the sole explanations for our findings [6], [13].

Analysis of other parameters using our analyzer showed increments of %-H-IPF and H-IPC which implies that immature platelets are capable of expansion ex-vivo. Alternatively, a less mature platelet fraction than H-IPF might exist in peripheral blood that replenishes H-IPF but cannot be detected by the analyzer used. However, increases in both PLCR and MPV at day 10 along with data obtained through TEM showing morphologically intact immature platelets in day 10 samples lend support to the ex-vivo expansion or the greater stability of this platelet fraction in prolonged storage. It will be of interest to determine if the immature platelets observed on day 10 samples are still functional and capable of giving origin to mature platelets. Along these lines, the optimal growth/preservation conditions required by immature platelets in storage need to be elucidated.

Ionizing irradiation is known to damage DNA and RNA and inhibit protein synthesis. However, ionizing irradiation of apheresis platelets has not been shown to have a clinically significant or demonstrably adverse effects on platelet function [14]–[16], which may be explained by the reduced DNA and RNA content in mature platelets. Our observations indicate that the %-IPF increment during storage is an active process that can be suppressed by irradiation, suggesting the presence of a biosynthetically active immature platelet population that depends on intact nucleic acids for protein production, function and longevity. Additionally, the presence of an actively proliferating IPF in platelet components might help maintain adequate platelet numbers in platelet components during storage.

How immature platelets give origin to mature ones is not understood but it has been shown that ex-vivo generated murine megakaryocytes infused into mice generate up to 200 platelets/cell in as little as 5 minutes [17]. The number of platelets derived from an immature platelet is not known but their isolation and use in similar experiments could begin to answer this. Since aging platelets undergo anuclear apoptotic cell death mediated by Bcl-x_L_ degradation that antagonizes the pro-apoptotic effect of Bak [18], similar mechanisms could also mediate immature platelets' lifespan.

Platelet transfusions prevent bleeding in patients with thrombocytopenia, but the clinical significance of %-IPF requires scrutiny. Animal studies have shown that newly formed murine immature platelets have increased hemostatic activity compared to mature ones as demonstrated by their increased response to thrombin and higher expression of surface P-selectin [19]. Since a small cohort of pediatric patients receiving peripheral blood stem cell transplantation transfused with %-IPF-high SDP units required fewer platelet transfusions and had fewer bleeding episodes [5], IPF may potentially improve the efficacy of platelet transfusions by sustaining platelet count in-vivo and mediate bleeding prevention. Larger clinical studies to determine the potential benefits of the immature platelet content in platelet products are needed. Our findings suggest that IPF may expand ex-vivo and have a longer shelf life than their mature counterparts. Further studies of IPF are of critical clinical importance due to the observed dynamics during storage.
